# Microhabitat Selection by Ground-Foraging Birds in Urban Parks

**DOI:** 10.3390/ani15081155

**Published:** 2025-04-17

**Authors:** Lucas M. Leveau

**Affiliations:** Departamento de Ecología, Genética y Evolución, Facultad de Ciencias Exactas y Naturales, Universidad de Buenos Aires—IEGEBA (CONICET-UBA), Intendente Güiraldes 2160, Ciudad Universitaria, Pab 2, Piso 4, Buenos Aires C1428EGA, Argentina; lucasleveau@yahoo.com.ar

**Keywords:** bird, feeding, green areas, behavior, habitat, urbanization

## Abstract

Urban parks are refuges for a diversity of birds in cities. Therefore, understanding the relationship between park structure and different bird species is essential to achieve nature-friendly park planning and management. However, the selection of foraging substrates by ground-feeding species has been very little explored. The aim of this study was to analyze the use and selection of foraging substrates by birds in urban parks in Buenos Aires City, Argentina. Between December 2020 and February 2021, 16 urban parks were visited. During each visit, focal observations of individuals feeding on different microhabitats were made. For ground-foraging species, the use of each substrate was then compared with its availability by means of contingency tests. A total of 38 species and 7823 foraging attempts were registered. The species with the most records were the Rufous-bellied Thrush (*Turdus rufiventris*), the Eared Dove (*Zenaida auriculata*), and the Rufous Hornero (*Furnarius rufus*). Most of the species foraged on ground substrates. Several species, such as the Thrush and the Picazuro Pigeon (*Patagioenas picazuro*), selected lawn and bare ground. The Monk Parakeet (*Myiopsitta monachus*) and the Cattle Tyrant (*Machetornis rixosa*) selected lawn, while the Eared Dove and the Picui Ground Dove (*Columbina picui*) selected bare ground. Some species such as the Picui Ground Dove and the Green-barred Woodpecker (*Colaptes melanochloros*) did not use impermeable surfaces. The results obtained revealed that not only is green cover necessary for birds in urban parks, but also other surfaces such as bare ground can favor native bird species.

## Simple Summary

Urban parks are refuges for a diversity of birds in cities. Therefore, understanding the relationship between park structure and different bird species is essential to achieve nature-friendly park planning and management. However, the selection of foraging substrates by ground-feeding species has been very little explored. The aim of this study was to analyze the use and selection of foraging substrates by birds in urban parks in Buenos Aires City, Argentina. The species with the most records were the Rufous-bellied Thrush (*Turdus rufiventris*), the Eared Dove (*Zenaida auriculata*), and the Rufous Hornero (*Furnarius rufus*). Most of the species foraged on ground substrates. Several species, such as the Thrush and the Picazuro Pigeon (*Patagioenas picazuro*), selected lawn and bare ground. The Monk Parakeet (*Myiopsitta monachus*) and the Cattle Tyrant (*Machetornis rixosa*) selected lawn, while the Eared Dove and the Picui Ground Dove (*Columbina picui*) selected bare ground. Some species such as the Picui Ground Dove and the Green-barred Woodpecker (*Colaptes melanochloros*) did not use impermeable surfaces. The results obtained revealed that not only is green cover necessary for birds in urban parks, but also other surfaces such as bare ground can favor native bird species.

## 1. Introduction

Urban parks are considered hot spots of bird diversity in urban areas [1]. Although park area seems to be the main variable explaining bird species richness in urban parks [2,3], other factors such as human disturbance, food and nesting resources availability, and habitat diversity and structure have also been proposed to affect bird species richness [4,5,6,7,8,9,10]. Several vegetation types may encourage the coexistence of bird species with different habitat requirements [11,12,13]. Information about bird use and selection of habitat components, or microhabitats, within parks is fundamental for bird conservation in urban areas. Whereas habitat use describes the actual distribution of individuals across habitat types [14,15], habitat selection is a behavioral response that may result in the disproportionate use of habitats [14,15,16]. In general, habitat selection may be analyzed by comparing habitat use by species with habitat availability [15].

Information about bird foraging microhabitat use and selection in urban parks is scarce and mainly focused on shrub or tree selection in urban parks located in forested biomes [17,18,19,20]. Studies that analyzed tree selection have found that bird species tend to select native trees for foraging [18,19]. However, in open or semi-open biomes where the bird species pool may be composed of a higher proportion of ground foragers, the bird foraging use and selection of ground substrates in urban parks may be significant and, thus, research is warranted. Ground substrates in urban parks are generally composed of lawns, bare ground, or concrete sidewalks, which may provide different availability of food resources. The objectives of this study were as follows: (1) to analyze the microhabitat foraging use of birds in urban parks, considering different components such as trees, shrubs, ground, or air; and (2) within the ground foragers, to analyze microhabitat foraging selection by comparing bird species use with the availability of ground substrates in urban parks. Because the study was conducted in a city surrounded by open or semi-open biomes, I expected to find a dominance of species foraging in ground substrates in urban parks. On the other hand, ground foragers were expected to select ground substrates, probably according to food availability.

## 2. Methods

**Figure 1:**
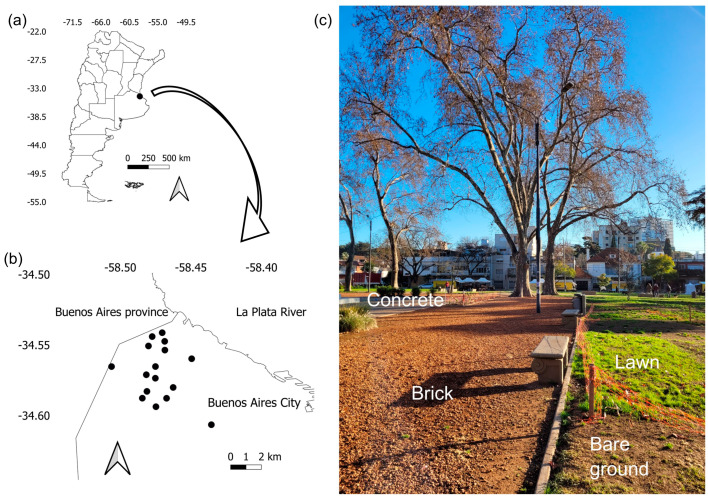
Location of Buenos Aires City in Argentina (**a**), location of the 16 urban parks (black dots) in Buenos Aires City (**b**), and example of urban park surveyed showing different types of ground substrates (**c**).

The study was carried out in urban parks of Buenos Aires City (34°35′59″ S, 58°22′55″ W; 25 masl; 31,206,121 inhabitants). Buenos Aires is located on the coast of La Plata River and is surrounded by crops, pastures, tree plantations, and scattered natural and semi-natural areas (Figure 1a,b). The native landscape was mainly composed of grasslands and xerophilous and deltaic forests [21,22].

A total of 16 parks were selected (Figure 1b), ranging from 0.3 to 14 Ha (mean = 3.52 Ha). Parks were separated by at least 500 m to ensure data independence. For example, the territory of a common species, the Rufous Hornero (*Furnarius rufus*), spans from 0.25 to 1 hectare [23]. In general, urban parks were composed of lawn areas, trees, shrubs, concrete or broken brick side sidewalks, bare ground, and playground areas (Figure 1c). Most tree species in urban parks are non-native, such as *Platanus acerifolia*, *Tilia viridis*, and *Morus alba*. Some species are non-native to the study area but are native to the northern part of Argentina, such as *Tipuana tipu* and *Ceiba speciosa*.

### 2.1. Bird Foraging Behavior

Each urban park was visited three times between December 2020 and February 2021, which coincides with the austral summer and breeding season of birds. During this period, many migrant species that nest in the study area and migrate to tropical areas in autumn are present, such as the Tropical Kingbird (*Tyrannus melancholicus*), the Gray-breasted Martin (*Progne chalybea*), and the Glittering-bellied Emerald (*Chlorostilbon lucidus*) [24]. Bird observations were made during the first four hours of the morning during days without rain or strong winds. Each park was walked through defined pathways, generally following trails, surveying its entire area with the help of binoculars. The time employed in each park varied in direct relation to its area. Birds foraging in all microhabitats of the park were surveyed. For example, different ground substrates included lawn, bare ground, concrete or brick sidewalks, playground areas, or sand areas. Tree substrates included foliage, branches, or trunks. Additionally, birds can forage in the air, defined as more than 2 m above substrates—for instance, species that engage in sally-strike foraging at heights greater than 2 m from the tree canopy, such as *Tyrannus melancholicus*, or species that hover at more than 2 m above the tree canopy or lawn, like the White-rumped Swallow (*Tachycineta leucorrhoa*) or the Brown-chested Martin (*Progne tapera*) [25]. Moreover, I noted whether species fed on fruits or leaves. Birds foraging on food provided directly or indirectly by humans were not considered.

During each sampling period, as many different birds as possible were observed. When an individual bird was sighted, sequential observations were made [26]. Sequential observations provide a more accurate description of the bird’s behavior than the recording of the initial behavioral observation [27]. Each bird individual was observed for up to 10 foraging attempts [28], and each foraging attempt was deemed as an independent foraging event [29]. While ground-foraging species were typically observed engaging in up to 10 foraging events, some individuals of species foraging in the canopy or air were seen making fewer than 10 attempts or until they were lost from sight. Although several authors pointed out that sequential observations of the same individual are not independent [29,30], I believe that my data obtained from several individuals in different parks helped to overcome this statistical issue.

### 2.2. Ground Substrate Characterization

Park microhabitat was measured by the location of random points. The number of points was proportional to park size. Parks smaller than 0.5 ha had one point, parks of 1 ha had two points, whereas parks larger than 2 ha had between three and eight points (Table S1). Four 20 m lines extended from the center toward each cardinal point, measuring the presence of each substrate cover every meter. Substrate covers were lawn, bare ground, concrete surfaces, playground areas, sidewalks of ground brick, and sand (Figure 1c). Then, the availability of each substrate was the proportion of presence in all points.

### 2.3. Statistical Analysis

The use of different park microhabitats by birds was analyzed through non-metric multidimensional scaling (NMDS), using a matrix with the proportional use of each microhabitat by species. NMDS is an ordination method that reduces data multidimensionality in a few axes, maintaining the order relationships among objects in the dissimilarity matrix [31]. A Bray–Curtis dissimilarity index was used. Only species with at least four records were analyzed. The NMDS was performed with the metaMDS function of the vegan package in R (version 4.4.2) [32,33].

The selection of ground substrates by bird species was analyzed through contingency tables. Only species with more than 50 foraging attempts were analyzed. Observed values of bird foraging in each substrate were compared with expected values, which were calculated by multiplying the proportion of each substrate with the total number of foraging events of each bird species. For those species with expected values equal or greater than 5, a Chi-square test was performed (*p* < 0.05) using the function chisq.test in R [33]. When expected values were lower than 5, a Fisher exact test was performed [34] using the fisher.test function in R [33].

## 3. Results

A total of 7787 observations of foraging behavior were made for 38 species (Table S2). Most of the observations corresponded to species that feed on plant material or seeds (45%) and invertebrates (32%) (Table S2). The species most observed were the Rufous-bellied Thrush (*Turdus rufiventris*) (18% of observations), the Eared Dove (*Zenaida auriculata*) (17%), and *Furnarius rufus* (13%).

**Figure 2:**
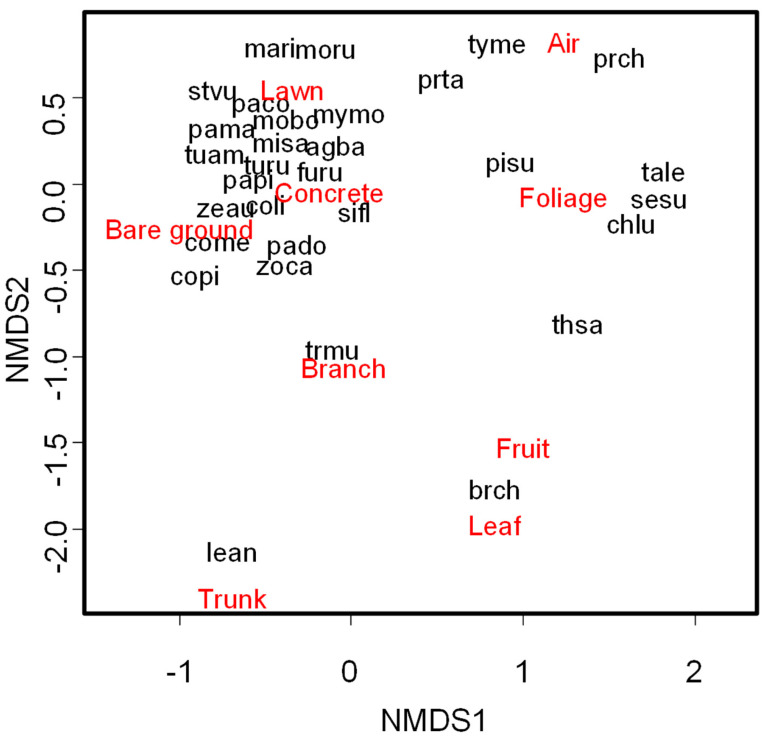
Non-metric multidimensional scaling (Stress = 0.09) showing the association between bird species and different habitat substrates and food resources (fruit, leaf) in urban parks of Buenos Aires City, Argentina. Brick and sand are aggregated to bare ground, whereas playground places are aggregated to concrete substrates. Only species with at least four records were analyzed. Species codes (alphabetical order): agba, *Agelaioides badius*; brch, *Brotogeris chiriri*; chlu, *Chlororstilbon lucidus*; coli, *Columba livia*; come, *Colaptes melanochloros*; copi, *Columbina picui*; furu, *Furnarius rufus*; lean, *Lepidocolaptes angustirostris*; mari, *Machetornis rixosa*; misa, *Mimus saturninus*; mobo, *Molothrus bonariensis*; moru, *Molothrus rufoaxillaris*; mymo, *Myiopsitta monachus*; paco, *Paroaria coronata*; pado, *Passer domesticus*; pama, *Patagioenas maculosa*; papi, *Patagioenas picazuro*; pisu, *Pitangus sulphuratus*; prch, *Progne chalybea*; prta, *Progne tapera*; sesu, *Serpophaga subcristata*; sifl, *Sicalis flaveola*; stvu, *Sturnus vulgaris*; tale, *Tachycineta leucorrhoa*; thsa, *Thraupis sayaca*; trmu, *Troglodytes musculus*; tuam, *Turdus amaurochalinus*; turu, *Turdus rufiventris*; tyme, *Tyrannus melancholicus*; zeau, *Zenaida auriculata*; zoca, *Zonotrichia capensis*. See English names in Table S2.

Most of the species observed were related to ground substrates, such as lawn, bare ground, or concrete (Figure 2). For example, the Monk Parakeet (*Myiopsitta monachus*) and the Red-crested Cardinal (*Paroaria coronata*) were associated with lawn, whereas the Eared Dove and the Green-barred Woodpecker (*Colaptes melanochloros*) were related to bare ground. The Rock Dove (*Columba livia*) was associated with concrete surfaces. On the other hand, a few species were associated with air or different shrub or tree substrates. For instance, *Tyrannus melancholicus* was seen foraging on the air, the Southern House Wren (*Troglodytes musculus*) was related to branches, and the Narrow-billed Woodcreeper (*Lepidocolaptes angustirostris*) was related to trunks (Figure 2).

The most common substrates of urban parks were lawn (39% of records), bare ground (13%), and concrete surfaces (12%). Other substrates such as sidewalks with brick, playground areas, and sand surfaces covered less than 3% of the ground.

**Figure 3:**
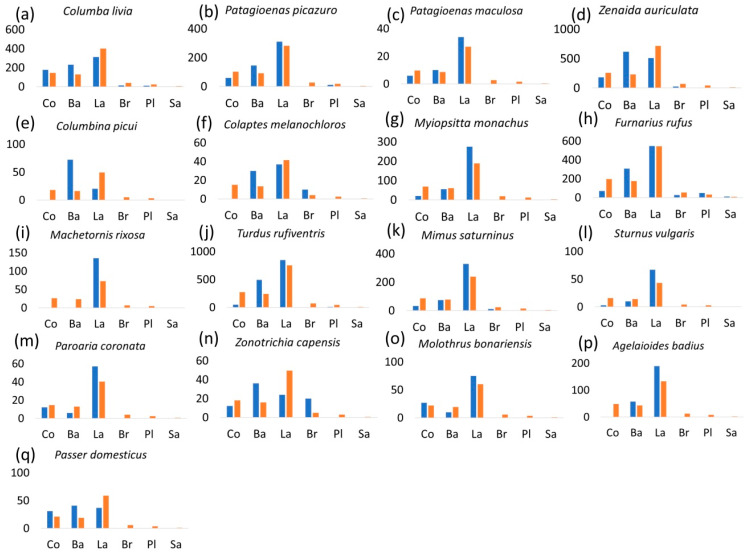
Number of observations using substrates (blue) and expected numbers of use (orange) by bird species with more than 50 observations in urban parks of Buenos Aires City, Argentina. All species except for *Patagioenas maculosa* (*p* = 0.151) exhibited significant differences between observed and expected values (*p* < 0.05; see Table 1). Co: concrete; Ba: bare ground; La: lawn; Br: brick; Pl: playground; Sa: sand.

Seventeen species with more than 50 foraging attempts were selected to analyze substrate selection (Table 1, Figure 3). All species, except the Spot-winged Pigeon (*Patagioenas maculosa,* Figure 3c), showed significant differences between substrate use and availability (Table 1). In general, there were four types of substrate selection: Firstly, bird species that selected lawn and bare ground substrates, such as the Picazuro Pigeon (*Patagioenas picazuro*), *Turdus rufiventris*, and the Grayish Baywing (*Agelaioides badius*) (Figure 3b,j,p). Secondly, bird species that mainly selected lawn areas, such as the Monk Parakeet (*Myiopsitta monachus*), the Cattle Tyrant (*Machetornis rixosa*)*,* the Chalk-browed Mockingbird (*Mimus saturninus*)*,* and the European Starling (*Sturnus vulgaris*) (Figure 3g,i,k,l). Thirdly, bird species that selected bare ground and concrete surfaces, such as *Columba livia* and the House Sparrow (*Passer domesticus*) (Figure 3a,q). Finally, a group of species that only selected bare ground, such as *Zenaida cunicularia*, the Picui Ground Dove (*Columbina picui*), and the Rufous-collared Sparrow (*Zonotrichia capensis*). Moreover, several species did not use concrete surfaces, such as *Columbina picui*, *Colaptes melanochloros*, *Machetornis rixosa*, and *Agelaioides badius*, although other species used concrete areas in a similar proportion to what was expected.

## 4. Discussion

The main findings indicated that most of the bird species in urban parks foraged on the ground, whereas a few bird species used tree substrates or the air. Within the guild of ground-foraging birds, most of the species selected different substrates, probably related to food availability or enhancing camouflage while foraging [35,36,37].

Most of the foraging species analyzed in this study were associated with ground substrates (65% of species, Figure 2). This result contrasts with other studies conducted in urban parks, where ground-foraging species in general comprised less than 50% of species or individuals [38,39,40,41,42,43,44,45]. The differences between studies may be related to the geographical location of cities and the species pool surrounding them [8]. Specifically, Buenos Aires is surrounded by agricultural areas, Pampa grasslands, and Talares (*Celtis tala*) light woodlands [24], where bird species typical of semi-open areas that forage on the ground numerically dominate communities [46,47,48] and have more chances to colonize urban parks. In contrast, the other studies of urban park bird communities [38,39,40,41,42,43,44] were conducted in cities surrounded by tropical or temperate forests, which probably favored the dispersion into urban parks of more bird species that forage in shrubs or trees. Therefore, assuming that the habitat structure of urban parks is homogeneous across studies due to common practices of design [49], the foraging guild structure of urban parks would be influenced by the regional pool of species. A similar pattern was found by Leveau et al. [50], where ground foragers were more common in cities surrounded by semi-arid scrubland than in cities surrounded by subtropical forests.

In general, the microhabitat use of the species in our study agrees with qualitative and quantitative assessments published [51,52,53,54,55,56,57]. Species known as ground foragers, such as *Turdus rufiventris* and *Agelaioides badius* [51,55], were mostly observed foraging in ground substrates in urban parks. According to Wilman et al. [57], *Turdus rufiventris* makes 100% use of the ground as a foraging substrate, whereas *Agelaioides badius* makes 50% use. In this study, *Turdus rufiventris* made more than 99% of ground use, which agrees with Wilman et al.’s [57] foraging use of ground. However, Gasperin and Aurélio Pizo [58] found a 66.8% ground use in an urban forest of Brazil. On the other hand, *Agelaioides badius* made 80% use of ground for foraging, differing from the estimations made by Wilman et al. [57]. These differences among studies may be due to regional variations in habitat structure or the existence of similar species competing for food, which could lead to changes in habitat use [59]. Therefore, my results highlight the importance of making foraging observations of birds in urban parks to obtain a priori guilds and give more accurate information about microhabitat use.

The results of microhabitat use should be interpreted cautiously because of potential differences in bird detectability. For instance, birds that use ground substrates might be easier to detect than those foraging within the tree canopy. Future studies should account for the differences in bird detectability across various microhabitats. In addition, bird species behavior can be influenced by the disturbance of pedestrians and dogs [60].

As expected, several species selected lawn areas. Lawns can provide food resources to species that eat plant material, such as *Patagioenas picazuro*, *Zenaida auriculata*, and *Myiopsitta monachus* [57,61,62]. In this regard, future studies should investigate how various lawn species influence the microhabitat selection of birds. On the other hand, *Myiopsitta monachus* can improve its camouflage with lawn due to its green plumage. Grey plumage species such as *Zenaida auriculata* and *Patagioenas picazuro* have more contrast with the green color of lawned areas, but they can improve predator vigilance by flocking together with *Myiopsitta monacha* (L. Leveau pers. obs.). Other species, such as *Machetornis rixosa, Sturnus vulgaris,* and *Mimus saturninus*, probably selected lawn substrates due to increased invertebrate availability [51,63].

Food availability and camouflage may promote the foraging selection of bare ground. For example, *Turdus rufiventris* may have more availability of earthworms in bare ground than in lawn areas. Additionally, their brown dorsal plumage enhances camouflage against the bare soil [36,64], aiding in evading predation from raptors like the Crested Caracara (*Caracara plancus*) and the Harris’s Hawk (*Parabuteo unicinctus*). *Zenaida auriculata* and *Columbina picui* can also be favored by increased food visibility, such as seeds, in bare ground and improved camouflage due to their grey plumage. In agreement with my results, Fontoura and Orsi [65] found higher use of soil substrates of *Zenaida auriculata* and *Columbina picui* in urban and agricultural areas of Brazil.

Exotic species, such as *Columba livia* and *Passer domesticus*, tended to select concrete surfaces for foraging, whereas most of the native bird species did not use or used less than expected concrete surfaces. Therefore, concrete surfaces should be avoided in urban parks for native bird conservation.

## 5. Conclusions

The results indicated that green cover is essential for birds in urban parks, while other surfaces like bare ground can benefit native bird species. Due to the increased visibility of food items, birds may prefer this type of ground substrate because it offers better food resource availability. Additionally, species with brown- or gray-colored backs can enhance their camouflage against raptors. More research is necessary to evaluate which mechanisms are behind bird substrate choice in urban parks.

## Figures and Tables

**Table 1:** Results of contingency table testing the independence between the number of foraging observations and the expected observations in different ground substrates. * Fisher exact test.

English Name	Scientific Name	Number of Individuals	Foraging Observations	Chi-Square	*p*
Rock Pigeon	*Columba livia*	82	743	66.44	<0.001
Picazuro Pigeon	*Patagioenas picazuro*	59	524	57.98	<0.001
Spot-winged Pigeon	*Patagioenas maculosa*	5	50	*	0.151
Eared Dove	*Zenaida auriculata*	138	1306	298.55	<0.001
Picui Ground Dove	*Columbina picui*	10	92	*	<0.001
Green-barred Woodpecker	*Colaptes melanochloros*	10	77	*	<0.001
Monk Parakeet	*Myiopsitta monachus*	38	350	74.43	<0.001
Rufous Hornero	*Furnarius rufus*	114	1008	109.11	<0.001
Cattle Tyrant	*Machetornis rixosa*	18	135	*	<0.001
Rufous-bellied Thrush	*Turdus rufiventris*	166	1402	345.01	<0.001
Chalk-browed Mockingbird	*Mimus saturninus*	54	444	61.5	<0.001
European Starling	*Sturnus vulgaris*	8	80	*	<0.001
Red-crested Cardinal	*Paroaria coronata*	8	75	*	0.012
Rufous-collared Sparrow	*Zonotrichia capensis*	11	92	*	<0.001
Shiny Cowbird	*Molothrus bonariensis*	12	112	*	0.003
Grayish Baywing	*Agelaioides badius*	12	247	79.78	<0.001
House Sparrow	*Passer domesticus*	11	109	*	<0.001

## Data Availability

Data will be available on request to the corresponding author.

## Supplementary Materials

The following supporting information can be downloaded at https://www.mdpi.com/article/10.3390/ani15081155/s1, Table S1: Location of the parks, area (ha), and number of points used to measure habitat characteristics. Table S2: List of species observed, including their foraging attempts and the percentage of total observations (N = 7787). Diet guilds based on Wilman et al. [57].
