# Supplementary material for: Microhabitat Selection by Ground-Foraging Birds in Urban Parks
Source: Animals (Basel). 2025 Apr 17;15(8):1155. doi: 10.3390/ani15081155 (PMC12023929; doi:10.3390/ani15081155)
Supplement: Supplementary file 1 [file animals-15-01155-s001.zip › animals-3519233-supplementary.pdf]

Table S1. Location of the parks, area (ha), and number of points used to measure habitat characteristics.

Park	Latitude	Longitude	Area	Number of points
1	-34.582926	-58.481131	0.32	1
2	-34.587752	-58.484401	0.34	1
3	-34.571127	-58.481562	0.47	1
4	-34.553676	-58.468413	0.64	1
5	-34.544092	-58.477406	0.77	1
6	-34.541342	-58.470299	0.82	1
7	-34.547452	-58.468537	0.86	1
8	-34.587803	-58.467215	1.14	2
9	-34.573596	-58.475161	1.21	2
10	-34.580138	-58.462534	1.31	2
11	-34.565322	-58.475085	1.4	2
12	-34.559783	-58.449424	5.32	3
13	-34.593801	-58.474744	7.36	4
14	-34.550741	-58.48004	9.53	4
15	-34.606545	-58.435465	14.89	4
16	-34.565348	-58.506212	19.96	8

Table S2. List of species observed, including their foraging attempts and the percentage of total observations (N= 7787). Diet guilds based on Wilman et al. [57].

English name	Scientific name	Diet	Foraging attempts	Percentage
Rock Pigeon	*Columba livia*	PlantSeed	743	9.54
Picazuro Pigeon	*Patagioenas picazuro*	PlantSeed	524	6.73
Spot-winged Pigeon	*Patagioenas maculosa*	PlantSeed	50	0.64
Eared Dove	*Zenaida auriculata*	PlantSeed	1306	16.77
Picui Ground-Dove	*Columbina picui*	PlantSeed	92	1.18
Green-barred Woodpecker	*Colaptes melanochloros*	Invertebrate	96	1.23
Checkered Woodpecker	*Dryobates mixtus*	Omnivore	20	0.26
Crested Caracara	*Caracara plancus*	VertFishScav	12	0.15
Turquoise-fronted Amazon	*Amazona aestiva*	Omnivore	25	0.32
Nanday Parakeet	*Aratinga nenday*	PlantSeed	11	0.14
Yellow-chevroned Parakeet	*Brotogeris chiriri*	FruiNect	53	0.68
Maroon-bellied Parakeet	*Pyrrhura frontalis*	PlantSeed	6	0.08
Monk Parakeet	*Myiopsitta monachus*	PlantSeed	470	6.04
Glittering-bellied Emerald	*Chlorostilbon lucidus*	FruiNect	51	0.65
Gilded Hummingbird	*Hylocharis chrysura*	FruiNect	12	0.15
Narrow-billed Woodcreeper	*Lepidocolaptes angustirostris*	Invertebrate	56	0.72
Rufous Hornero	*Furnarius rufus*	Invertebrate	1028	13.20
Cattle Tyrant	*Machetornis rixosa*	Invertebrate	137	1.76
Tropical Kingbird	*Tyrannus melancholicus*	Invertebrate	12	0.15
White-crested Tyrannulet	*Serpophaga subcristata*	Invertebrate	32	0.41
Great Kiskadee	*Pitangus sulphuratus*	Omnivore	21	0.27
White-rumped Swallow	*Tachycineta leucorrhoa*	Invertebrate	31	0.40
Gray-breasted Martin	*Progne chalybea*	Invertebrate	44	0.57
Brown-chested Martin	*Progne tapera*	Invertebrate	39	0.50
Rufous-bellied Thrush	*Turdus rufiventris*	Omnivore	1385	17.79
Creamy-bellied Thrush	*Turdus amaurochalinus*	Omnivore	52	0.67
Southern House Wren	*Troglodytes musculus*	Invertebrate	77	0.99
Chalk-browed Mockingbird	*Mimus saturninus*	Invertebrate	446	5.73
Sayaca Tanager	*Thraupis sayaca*	Omnivore	25	0.32
Saffron Finch	*Sicalis flaveola*	PlantSeed	60	0.77
Red-crested Cardinal	*Paroaria coronata*	Invertebrate	78	1.00
Rufous-collared Sparrow	*Zonotrichia capensis*	PlantSeed	102	1.31
Variable Oriole	*Icterus pyrrhopterus*	Invertebrate	21	0.27
Grayish Baywing	*Agelaioides badius*	Invertebrate	308	3.96
Screaming Cowbird	*Molothrus rufoaxillaris*	PlantSeed	42	0.54
Shiny Cowbird	*Molothrus bonariensis*	Invertebrate	117	1.50
House Sparrow	*Passer domesticus*	PlantSeed	123	1.58
European Starling	*Sturnus vulgaris*	Omnivore	80	1.03
